# Effects of Different Short-Term UV-B Radiation Intensities on Metabolic Characteristics of *Porphyra haitanensis*

**DOI:** 10.3390/ijms22042180

**Published:** 2021-02-22

**Authors:** Shimei Fu, Song Xue, Jun Chen, Shuai Shang, Hui Xiao, Yu Zang, Xuexi Tang

**Affiliations:** 1 College of Marine Life Sciences, Ocean University of China, Qingdao 266000, China; fushimei@stu.ouc.edu.cn (S.F.); xuesong@stu.ouc.edu.cn (S.X.); chenjun@ouc.edu.cn (J.C.); xiaohui@ouc.edu.cn (H.X.); 2 College of Biological and Environmental Engineering, Binzhou University, Binzhou 256600, China; shangshuai8983@126.com; 3 Laboratory for Marine Ecology and Environmental Science, Qingdao National Laboratory for Marine Science and Technology, Qingdao 266000, China; 4 Key Laboratory of Marine Eco-Environmental Science and Technology, First Institute of Oceanography, Ministry of Natural Resources, Qingdao 266000, China

**Keywords:** UV-B radiation, metabonomics, growth, amino acid metabolism, carbohydrate metabolism, UV-absorbing substances, adaptive response, *Porphyra haitanensis*

## Abstract

The effects of ultraviolet (UV) radiation, particularly UV-B on algae, have become an important issue as human-caused depletion of the protecting ozone layer has been reported. In this study, the effects of different short-term UV-B radiation on the growth, physiology, and metabolism of *Porphyra haitanensis* were examined. The growth of *P. haitanensis* decreased, and the bleaching phenomenon occurred in the thalli. The contents of total amino acids, soluble sugar, total protein, and mycosporine-like amino acids (MAAs) increased under different UV-B radiation intensities. The metabolic profiles of *P. haitanensis* differed between the control and UV-B radiation-treated groups. Most of the differential metabolites in *P. haitanensis* were significantly upregulated under UV-B exposure. Short-term enhanced UV-B irradiation significantly affected amino acid metabolism, carbohydrate metabolism, glutathione metabolism, and phenylpropane biosynthesis. The contents of phenylalanine, tyrosine, threonine, and serine were increased, suggesting that amino acid metabolism can promote the synthesis of UV-absorbing substances (such as phenols and MAAs) by providing precursor substances. The contents of sucrose, D-glucose-6-phosphate, and beta-D-fructose-6-phosphate were increased, suggesting that carbohydrate metabolism contributes to maintain energy supply for metabolic activity in response to UV-B exposure. Meanwhile, dehydroascorbic acid (DHA) was also significantly upregulated, denoting effective activation of the antioxidant system. To some extent, these results provide metabolic insights into the adaptive response mechanism of *P. haitanensis* to short-term enhanced UV-B radiation.

## 1. Introduction

Anthropogenic pollution of the atmosphere due to rapid industrialization in the past few decades caused an increase in pollutants that is responsible for the depletion of the ultraviolet (UV)-screening ozone layer in the stratosphere [1,2]. Therefore, UV radiation in the biologically relevant wavebands of UV-B (280–315 nm), especially its harmful effects on terrestrial and marine living organisms, became an important issue over the past decades [3]. In general, UV-B can penetrate down to a water depth of 20–30 m in the marine realm [4], and it may reach depths of 70 m in clear ocean [5]. The biological consequences of changes toward higher intensity of UV-B radiation in marine ecosystems are not fully understood. One question is whether UV-B radiation reaches organisms in their habitats.

In contrast to phytoplankton, macroalgae are attached to the substratum in the intertidal as well as upper subtidal zones and are directly exposed to the highest levels of UV-B radiation with the change of tides [6]. Tidal exposure imposes considerable environmental stress on intertidal seaweeds, such as different irradiance levels [7,8,9] and temperature changes [10,11,12]. Because UV-radiation daily doses in the intertidal system are much higher than in the sublittoral zone, there is a correlation between UV radiation tolerance and the vertical distribution of intertidal macroalgae [3,13]. 

There have been numerous studies on the harmful effects of UV-B radiation on algae, which include decreased performance or death. In seaweeds, studies have focused on the effects of UV-B radiation on growth [14,15] and development [16], productivity [17], photosynthesis [18,19,20], antioxidant systems [21,22], nutrition quality [23,24], and DNA damage [25,26]. Over time, seaweeds may develop a wide range of strategies to cope with UV radiation, such as absorbing screening compounds including carotenoids [27], mycosporine-like amino acids (MAAs) [28,29], phenols [30] and proteins [31] and repairing enzymes [32] that enable adaptation to environmental stress. The effects of UV-B radiation on seaweeds in natural conditions are complex. The adaptability of seaweeds to an increase in UV-B radiation is dictated by a complex metabolic activity involving protection, repair, and other factors [33]. 

Changes in the concentrations of metabolites detected in metabolic analysis can provide an overall understanding of the entire stress response [34]. Metabolite profiling is an important tool for characterizing the metabolic status of a plant with respect to environmental and developmental factors and represents the amplification and integration of signals from other functional genomic levels (e.g., transcriptome and proteome) [35].

*Porphyra haitanensis* (Bangiales, Rhodophyta) is the principal seaweed species that has been cultivated in the intertidal zones of South China coastlines [36]. It is an important economic cultivation seaweed [37] that contains high amounts of bioactive metabolites, including amino acids, polysaccharides, and minerals, and is thus widely consumed in world [38,39]. The production and quality of the cultivated *P. haitanensis* thalli are frequently influenced by intertidal environmental stress. Tidal exposure imposes large daily changes in light irradiance, and *P. haitanensis* is more vulnerable to direct exposure from UV-B radiation. The aims of the present study were to explore the physiological and metabolic response of intertidal macroalgae to UV-B radiation. We selected *P. haitanensis* as a model to characterize changes in growth, physiology, and metabolomic profile under short-term enhanced UV-B radiation. In addition, the key metabolic pathways involved in stress response were screened out to elucidate the regulatory mechanism by which macroalgae adapt to UV-B radiation.

## 2. Results

### 2.1. Effects of UV-B Radiation on the Morphology and Relative Growth Rate of P. haitanensis

**Figure 1:**
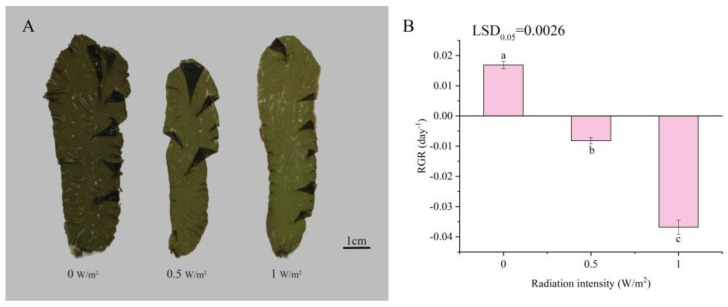
Changes in the morphology (**A**) and relative growth rate (**B**) of *Porphyra haitanensis* under different UV-B radiation intensities. Error bars are standard deviations (*n* = 4). Columns with different letters are statistically different (*p* < 0.05).

The tolerance limit of *P. haitanensis* to UV-B exposure was evaluated based on the morphology and growth after exposure to UV-B radiation. With increasing UV-B radiation intensity, the color of thalli gradually became lighter compared with the controls (Figure 1A). The bleaching phenomenon in the 1 W/m^2^ UV-B treatment was more obvious. Compared with the control, the relative growth rate of *P. haitanensis* significantly decreased under different UV-B radiation intensities (*p* < 0.05). The relative growth rate (RGR) of the algae began to turn negative at 0.5 W/m^2^ and decreased to −0.0368 in the 1 W/m^2^ UV-B treatment (Figure 1B).

### 2.2. Effects of UV-B Irradiation on Total Amino Acids, Soluble Sugar, Total Protein, and MAAs of P. haitanensis

**Figure 2:**
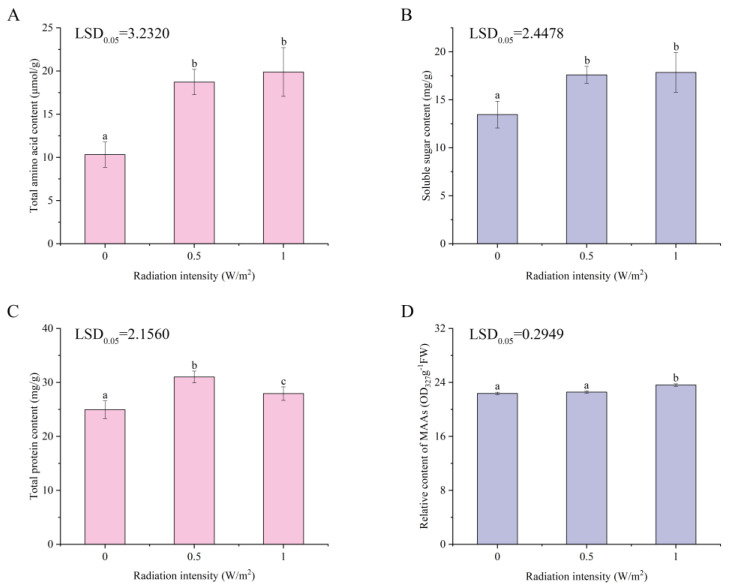
Changes in total amino acids (**A**), soluble sugar (**B**), total protein (**C**), and MAAs (**D**) content in *Porphyra haitanensis* under different UV-B radiation intensities. Error bars are standard deviations (*n* = 4). Columns with different letters are statistically different (*p* < 0.05).

Total amino acid, soluble sugar, total protein, and MAAs contents of UV-B-treated and untreated thallus were examined after 2 days of UV-B treatment. Total amino acid (Figure 2A) and soluble sugar (Figure 2B) contents were significantly increased at 0–0.5 W/m^2^ UV-B (*p* < 0.05) and slightly increased at 0.5–1 W/m^2^ UV-B (*p* > 0.05). Compared with the control, total protein (Figure 2C) content significantly increased under different UV-B radiation intensities (*p* < 0.05). The MAAs (Figure 2D) content slightly increased at 0–0.5 W/m^2^ UV-B (*p* > 0.05), followed by a marked increase at 0.5–1 W/m^2^ (*p* < 0.05).

### 2.3. Analysis of Differential Metabolites of P. haitanensis under Different UV-B Radiation Intensities

To understand the metabolic response regulation of *P. haitanensis* to UV-B exposure, we identified metabolites showing significant changes in abundance after 2 days of UV-B treatment by ultra-high performance liquid chromatography quadrupole time-of-flight mass spectrometer (UHPLC-Q-TOF MS ) analysis. Through principal component analysis (PCA), samples within the same group were clustered together in either positive or negative ion mode, with obvious separation between groups and without intersection phenomenon (Supplementary Figure S1 and Supplementary Tables S1 and S2). These findings indicate significant differences in metabolic regulation exposure to different UV-B radiation intensities, demonstrating that metabolites of *P. haitanensis* were significantly affected by UV-B radiation.

**Figure 3:**
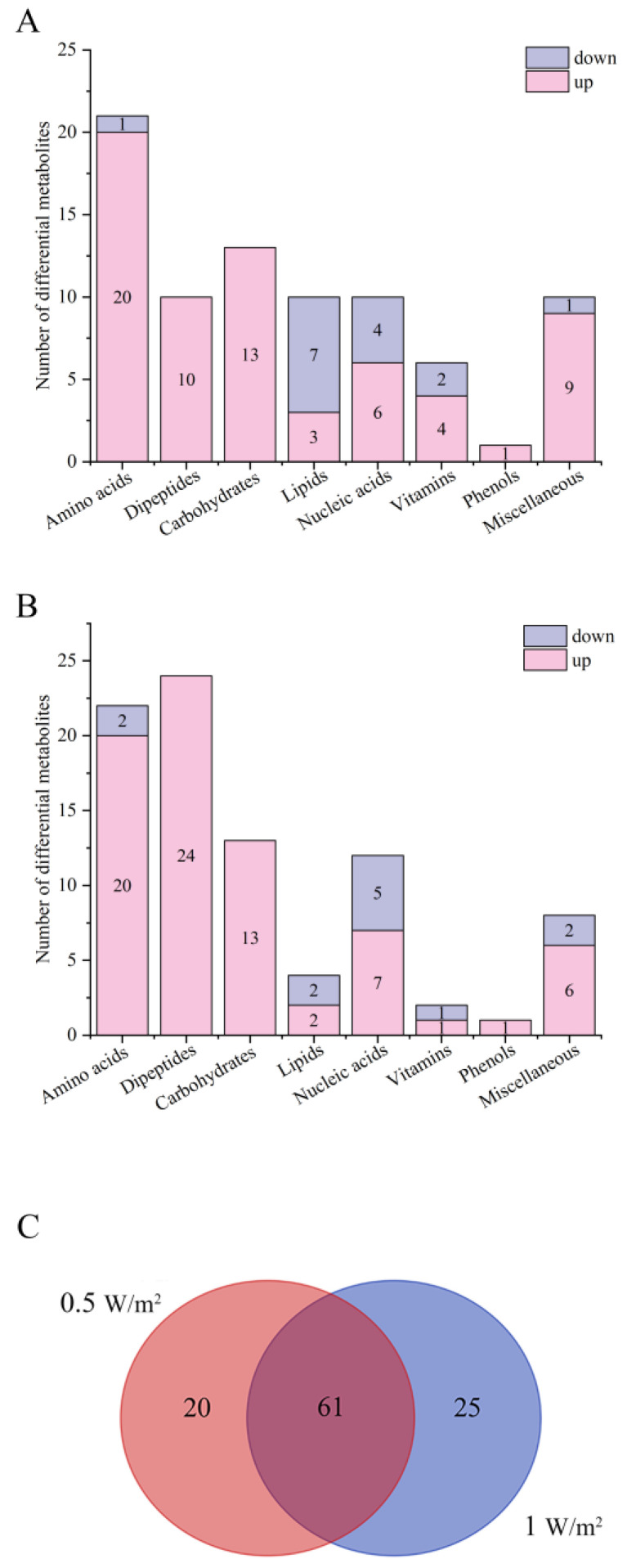
Metabolite analysis of *Porphyra haitanensis* under different UV-B radiation intensities. (**A**) Differential metabolites in the 0.5W/m^2^ UV-B treatment compared with the control; (**B**) differential metabolites in the 1W/m^2^ UV-B treatment compared with the control; and (**C**) Venn diagram of differential metabolites under different UV-B radiation intensities.

Compared with the control, 81 and 86 metabolites were significantly changed in the 0.5 W/m^2^ and 1 W/m^2^ UV-B treatments (variable importance in the projection (VIP) > 1, *p* < 0.05), respectively, mainly including amino acids, dipeptides, carbohydrates, lipids, nucleic acids, vitamins, and phenols (Figure 3A,B). Detailed information regarding these metabolites, including their taxonomical characterization are summarized in Supplementary Table S3. Compared with the control, 66 were upregulated and 15 were downregulated in the 0.5 W/m^2^ UV-B treatment, while 74 were upregulated and 12 were downregulated in the 1 W/m^2^ UV-B treatment. The differential metabolites of *P. haitanensis* were mainly upregulated and primarily composed of amino acids and carbohydrates. The number of upregulated differential metabolites in the 1 W/m^2^ UV-B treatment was the highest, accounting for 86.05% of the total differential metabolites within the group.

**Figure 4:**
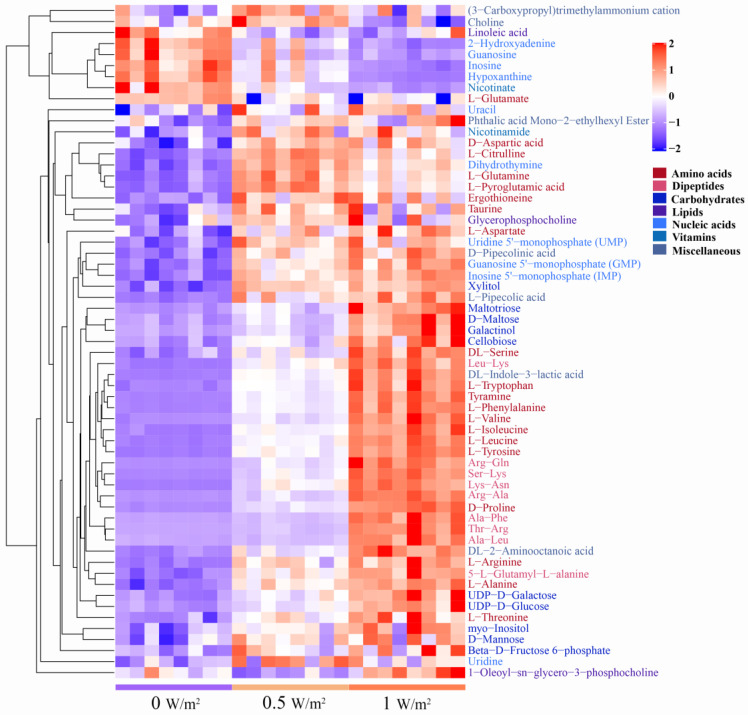
Clustering analysis of differential metabolites of *Porphyra haitanensis* under different UV-B radiation intensities. Rows and columns represent metabolites and treatment groups, respectively. The red color indicates a high abundance of a metabolite, whereas the blue color represents a low relative abundance of a metabolite. Name of metabolites are marked in different colors to differentiate chemical taxonomy.

There were 61 identical differential metabolites in the 0.5 W/m^2^ and 1 W/m^2^ UV-B treatment (Figure 3C), and most of the differential metabolites (about 95.08%) showed similar trends, including a significant increase in 51 metabolites and a significant decrease in 7 metabolites in both comparison groups (Figure 4, Table S4). These metabolites were mainly divided into two categories by hierarchical clustering analysis (Figure 4). Compared with the control, the first category was significantly upregulated under different UV-B radiation intensities, including most amino acids, carbohydrates, and lipids, while the content of the second category decreased with increasing UV-B intensity, including guanosine, hypoxanthine, and linoleic acid.

### 2.4. Metabolic Pathway Analysis of Differential Metabolites of P. haitanensis under Different UV-B Radiation Intensities

**Figure 5:**
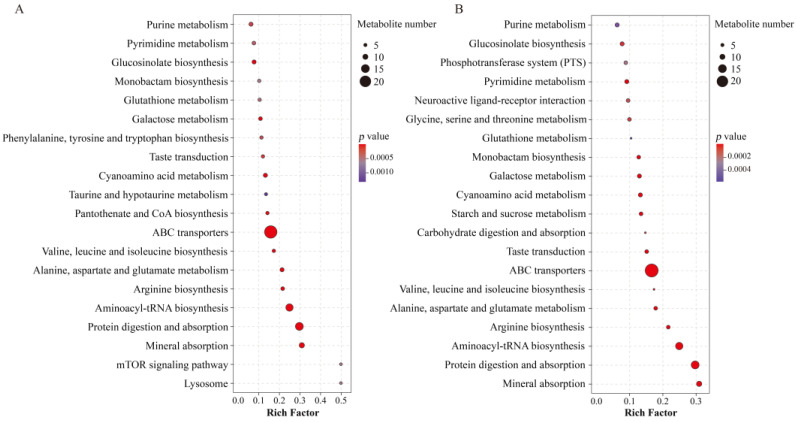
Bubble diagram of metabolic pathway analysis of *Porphyra haitanensis* in the 0.5 W/m^2^ (**A**) and 1 W/m^2^ (**B**) UV-B treatments compared with the control. The shade of the dot color indicates the size of *p* value, which represents the significance of the degree of influence on a certain pathway. The red color indicates that the smaller the *p* value, the more significant the effect was on the pathway. The purple color means higher *p* value. A larger area of the point indicates that more differential metabolites were involved in the metabolic pathway. The figure shows the first 20 metabolic pathways in the enrichment metabolic pathway with a *p* value < 0.05.

The relevant metabolic pathways are shown in Figure 5. Among these, amino acid metabolism, carbohydrate metabolism, and glutathione metabolism changed significantly after different UV-B radiation treatment.

**Figure 6:**
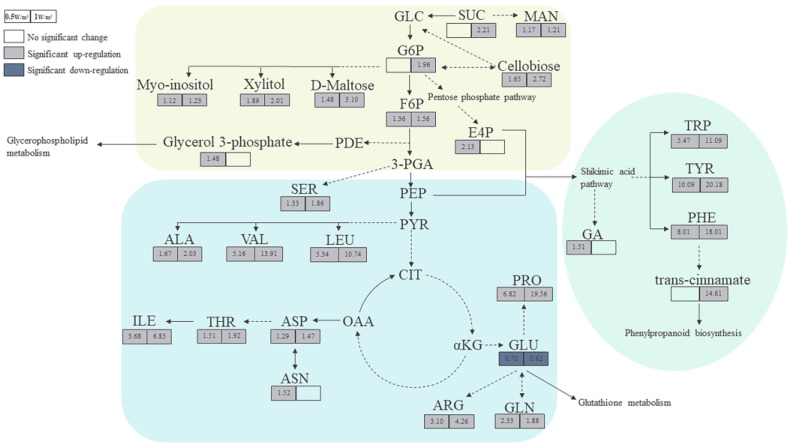
Metabolic pathway of *Porphyra haitanensis* under different UV-B radiation intensities. The values in the box represent the fold change of the metabolites; a multiple > 1 indicates upregulation, while a multiple < 1 indicates downregulation. The black solid and dotted arrows indicate direct and indirect reactions, respectively. MAN, D-mannose; SUC, sucrose; GLC, glucose; G6P, D-glucose 6-phosphate; F6P, beta-D-fructose-6-phosphate; PDE, dihydroxyacetone phosphate; 3-PGA, glycerate-3-phosphate; SER, DL-serine; PEP, phosphoenolpyruvic acid; E4P, D-erythrose 4-phosphate; GA, gallic acid; PHE, L-phenylalanine; TYR, L-tyrosine; TRP, L-tryptophan; PYR, pyruvic acid; ALA, L-alanine; VAL, L-valine; LEU, L-leucine; CIT, citric acid; OAA, oxaloacetic acid; ASP, L-aspartate; ASN, L-asparagine; THR, L-threonine; ILE, L-isoleucine; αKG, α-ketoglutarate; GLU, L-glutamate; GLN, L-glutamine; ARG, L-arginine; PRO, D-proline.

To more intuitively investigate the metabolic response of *P. haitanensis* to UV-B radiation, changes in target differential metabolites and related pathways were compared (Figure 6). The contents of most amino acids in *P. haitanensis* significantly increased under different UV-B radiation intensities, except for glutamate, which continued to be significantly downregulated. Compared with the control, the upregulation range of leucine, valine, isoleucine, phenylalanine, arginine, tyrosine, tryptophan, and proline increased by over 3.10-fold in different UV-B radiation treatments. Monosaccharides (such as mannose, beta-D-fructose-6-phosphate, D-glucose-6-phosphate, and D-erythrose-4-phosphate), oligosaccharides (such as sucrose, cellobiose, and maltose), and sugar alcohols (such as myo-inositol and xylitol) were significantly upregulated under different UV-B radiation treatments. Among these, D-erythrose-4-phosphate significantly increased only in the 0.5 W/m^2^ UV-B treatment; sucrose and glucose-6-phosphate significantly increased only in the 1 W/m^2^ UV-B treatment. These carbohydrates are mainly involved in galactose metabolism, starch and sucrose metabolism. Compared with the control, the content of phenolic substances (such as gallic acid) significantly increased with the 0.5 W/m^2^ UV-B treatment, and cinnamic acid, a key substance in the phenylpropane synthesis pathway, was significantly upregulated with the 1 W/m^2^ UV-B treatment.

## 3. Discussion

Because intertidal macroalgae have to face naturally high UV-B irradiation due to their habitats, they have adapted a set of protective mechanisms allowing survival in the intertidal zone. The effects of enhanced UV-B radiation on the growth, morphology, physiology, and metabolism of algae can help to understand what protective strategies will be taken against solar UV-B stress. Here, short-term enhanced UV-B radiation inhibited *P. haitanensis* growth, and the bleaching phenomenon occurred in the thalli. However, most of the amino acids, carbohydrates, and phenols were significantly upregulated under UV-B exposure. These observed changes in metabolites under short-term enhanced UV-B indicate that the *P. haitanensis* regulates the synthesis/degradation of these metabolites or the activity of related metabolic pathways to protect against UV-B radiation damage.

The negative effects on growth and development caused by UV-B irradiation are well documented, and usually relative growth rates (RGRs) are also related to UV damage to the photosynthetic machinery and photosynthetic pigments caused by increasing UV radiation [40]. In this study, with increasing UV-B radiation intensity, the RGR of *P. haitanensis* significantly decreased, thalli became lighter in color, and the bleaching phenomenon occurred. Similarly, *Ulva expansa* (Setch.) S. and G. (Chlorophyta) thalli showed significantly lower growth rates after exposure to UV-B radiation [41]. In addition, UV-B treatment might cause the wastage of photosynthetic pigments in marine macroalgae [20]. These observations indicate that UV-B irradiation destroys photosynthetic pigments and impairs photosynthesis, further inhibiting growth of algae.

Amino acids are not only involved in protein biosynthesis, but also act as precursors of related metabolites in response to environmental stress [42]. The contents of amino acids and total protein in organisms significantly increased to resist stress [43,44]. Proline can counteract stress damage through cellular osmotic regulation, removal of reactive oxygen species, and protective membrane integrity [45]. Alanine is synthesized by alanine aminotransferases from pyruvate, and thus is closely related to glycolysis and the TCA cycle [46]. The significant increase in alanine content reflects the positive response of these energy metabolic pathways. As a branched-chain amino acid, valine could make significant negative contributions to reactive oxygen species (ROS) generation [47]. Besides, it also immediately and indirectly participates in many crucial metabolic functions, including the biosynthesis of aminoacyl-tRNAs, which are components of the protein synthesis machinery [48]. Aromatic amino acids (such as tyrosine, phenylalanine, and tryptophan) could absorb UV-B radiation; therefore, these are more vulnerable to the direct effects of UV-radiation [49,50]. Glutamate is an important substrate for amino acid metabolism and is used for the synthesis of other amino acids; in consequence, the content of glutamate tends to decrease under UV-B radiation [51]. These are consistent with the results of this study. The contents of total amino acids and protein in *P. haitanensis* significantly increased with UV-B radiation treatment (Figure 2A,C). Regarding individual amino acids, except for glutamic acid, the contents of most amino acids significantly increased.

Moreover, tyrosine and phenylalanine are important precursors of the phenylpropanoid biosynthesis pathway, and phenols synthesized through this pathway can absorb UV-B radiation and protect organisms from radiation [52,53]. Tyrosine, phenylalanine, and phenolic substances (gallic acid) in *P. haitanensis* were significantly upregulated under UV-B radiation. In addition, cinnamic acid, a key intermediate of the phenylpropanoid biosynthesis pathway, showed a significant increase in content with 1 W/m^2^ UV-B treatment. Most algae synthesize MAAs as intracellular UV-sunscreens that could emit the absorbed ultraviolet radiation energy as heat to prevent UV-induced photodamage [27]. Studies have shown that ultraviolet radiation can promote the synthesis of MAAs in intertidal macroalgae [54,55]. MAAs contained in algae include porphyra-334, shinorine, and euhalothece-362, which are synthesized by threonine, serine, and alanine, respectively [56]. Intriguingly, the induction of MAAs (Figure 2D) was in connection with a marked increase of amino acids including threonine, serine, and alanine. These results demonstrate that *P. haitanensis* can synthesize UV-absorbing substances (such as phenols and MAAs) through amino acids to adapt to enhanced UV-B radiation. 

Carbohydrates are not only the main energy substances in organisms, but they also play an important regulatory role in plant growth, development, and stress response as signaling molecules [57,58]. The previous study indicated that the accumulation of soluble sugar (mainly sucrose) could increase the tolerance of plants to abiotic stress [59] and contribute to the osmotic regulation of cells [60]. It is believed that the catabolism of glucose also provides energy for plant growth and reduces oxidative damage [61]. Sugar alcohols could either act as intermediates in redox reactions [59] or stand as true ROS scavengers through the hydrogen atom transfer mechanism [62] to resist oxidative stress. Inositol is a signaling molecule [63] that may play an important role in signal transduction in response to stress. Different UV-B radiation treatments significantly increased the content of total soluble sugar in *P. haitanensis* (Figure 2B), which is similar to the trend of upregulation and accumulation of carbohydrates detected by metabonomic techniques. The present research has shown that the contents of sucrose, glucose metabolites (D-glucose 6-phosphate and beta-D-fructose-6-phosphate), pentose phosphate pathway product (D-erythrose-4-phosphate), and sugar alcohols (myo-inositol and xylitol), as well as cell wall-related metabolites (cellobiose) and sugar storage forms (D-maltose), significantly increased under UV-B exposure. The results indicate that carbohydrate metabolism in *P. haitanensis* responds positively under different UV-B radiation intensities by providing ATP and removing free radicals. 

Glutathione metabolism helps maintain the redox state of cells [64]. Ascorbic acid/ dehydroascorbic acid (DHA) in this metabolic pathway plays an important role in reducing stress-induced oxidative damage as an important redox pair [65,66,67]. In this study, DHA was also significantly upregulated in *P. haitanensis*. It was previously shown that the activity of antioxidants was significantly enhanced in *Ulva fasciata* against UV-B-induced oxidative stress, including a significant accumulation of DHA [22]. This may be due to the fact that ascorbate is the most important reducing substrate for H_2_O_2_ detoxification, which enhances the antioxidant capacity of the algae and resists UV-B stress [65]. 

## 4. Materials and Methods

### 4.1. Algal Materials and Growth Conditions

Thalli of *P. haitanensis* were collected in November 2019 from the intertidal regions at Pingtan (119.77° E, 25.42° N), Fujian Province, China. After harvesting, whole algae were thoroughly washed with natural seawater to remove any attached sand and immediately dried in the dark and stored at −20 °C until culture. The thalli were softly rinsed with sterilized seawater to prevent microbial contamination and then pre-incubated at 20 ± 0.5 °C for 3 days in aerated seawater with sterilized Provasoli’s enrichment solution medium (PES). The algae received irradiance of about 50 µmoL photons·m^−2^·s^−1^ (PAR) under a 12 h/12 h of light/dark period. The seawater was sterilized and continuously renewed every day.

### 4.2. UV-B Treatments 

After renaturation, healthy thalli with similar shape were transferred to a plastic square plate (inner diameter: length, 23 cm; width, 17 cm; height, 4.5 cm) containing 300 mL of culture seawater. UV-B radiation (280–320 nm) was applied using UV-B lamps (Philips, TL 40W/12 RS, Hamburg, Germany) wrapped with 0.08 mm thick cellulose diacetate film (to prevent UV-C radiation below 290 nm) under 50 μmol photons·m^−2^·s^−1^ (PAR) provided by cool-fluorescent lamps (Philips, TL5-28W, Shanghai, China).

UV-B radiation intensity was determined by adjusting the distance between UV-B lamps and the thalli and measured by an UV-B radiometer (Photoelectric Instrument Factory of Beijing Normal University, China). The UV-B experiment in this study involved three treatments: control (0 W/m^2^) and 0.5 W/m^2^ and 1 W/m^2^ of UV-B radiation for 1 h each day for 2 days.

### 4.3. Physiological Analysis 

#### 4.3.1. Relative Growth Rate

The algal samples were gently blotted on paper towels to remove excess water before weighing. Changes in algal wet biomass of different UV-B-treated groups were measured to estimate growth. The relative growth rate (RGR) of the algae was calculated according to the following formula [68]:

(1) $${\mathrm{RGR}(\mathrm{day}}^{-1})=\frac{{\mathrm{lnW}}_{2}{-\mathrm{lnW}}_{1}}{t_{2}{-t}_{1}}$$

where W_1_ is the initial fresh mass (g), W_2_ is the final fresh mass (g), t_1_ represents the initial time, and t_2_ is the final time of the experiment. 

#### 4.3.2. Total Amino Acids, Soluble Sugar, Total Protein, and MAAs

Total amino acids were measured using an amino acid (AA) content assay kit (Beijing Solarbio Science & Technology Co., Ltd. Beijing, China). The α-amino of amino acids reacts with hydrated ninhydrin to produce a blue-purple compound with a characteristic absorption peak at 570 nm. Amino acid content was calculated by measuring absorbance at 570 nm. 

Soluble sugar was determined using a plant-soluble sugar content assay kit (Beijing Solarbio Science & Technology Co., Ltd. Beijing, China). The detection principle involves the anthrone colorimetric method. Soluble sugar content was calculated by measuring absorbance at a wavelength of 620 nm.

The total protein content was determined using a protein quantitative kit (Nanjing Jiancheng Bioengineering Institute, Nanjing, China). Under alkaline conditions, the protein reduces Cu^2+^ to Cu^+^, and Cu^+^ reacts with the BCA (bicinchoninic acid) reagent to form a purple complex with a maximum absorption peak of 562 nm. The content of total protein can be calculated by measuring the absorbance at 562 nm. 

The MAAs extraction method referred to Scherer et al. [69]. Fresh thalli (0.1 g) were ground, then received 10 mL of 25% (volume fraction) methanol, incubated in a 60 °C water bath for 15 min, cooled to room temperature, and centrifuged for 15 min (8000 r/min). MAAs have absorption maxima in the wavelength range of 320–360 nm [29]. Therefore, the absorption of the supernatants in the range of 250–800 nm was measured using an ultraviolet spectrophotometer (UV-8000, Shanghai, China), and the maximum absorption peak was found at 327 nm. The content of MAAs was estimated by recording the absorbance value at 327 nm, and the result was expressed as OD_327_ g^−1^ FW (fresh weight).

The results of physiological indexes were expressed as the mean ± standard deviation of four replicates. Data were analyzed using IBM SPSS Statistics 25 with one-way ANOVA, followed by Least Significant Difference (LSD) to determine whether they were significantly different at the 0.05 probability level (*p* < 0.05). Calculation method of LSD_0.05_ value referred to Wang et al. [70]. 

### 4.4. Metabolic Analysis and Data Processing

At the end of the second day of UV-B treatment, the thalli of *P. haitanensis* were immediately frozen in liquid nitrogen and stored at −80 °C. Eight biological replicates (80 mg per replicate) were selected for metabolic analysis in each treatment group. Approximately 80 mg of thalli were quickly weighed after grinding in liquid nitrogen, respectively mixed with 200 μL water for homogenization, vortexed for 60 s, received 800 μL of methanol acetonitrile solution (1:1, *v*/*v*), vortexed for 60 s, ultrasonicated twice at low temperature for 30 min, and placed at −20 °C for 1 h to precipitate proteins. Following this, the mixtures were centrifuged at 14,000 rcf (relative centrifugal force) for 20 min (4 °C), and then the supernatants were collected, freeze-dried, and stored at −80 °C.

The samples were separated by an Agilent 1290 Infinity LC ultra-high performance liquid chromatography (UHPLC) system and a hydrophilic interaction liquid chromatography (HILIC) column, and the conditions were as follows: column temperature, 25 °C; flow rate, 0.3 mL/min; injection volume, 2 μL. To avoid the influence of signal fluctuation of instrument detection, continuous analysis of samples was performed in random. The quality control (QC) samples were inserted into the sample queue to monitor and evaluate the stability of the system and the reliability of the experimental data. Electrospray ionization (ESI) positive ion and negative ion modes were used for detection. The samples were analyzed with mass spectrometry by a Triple TOF 5600 mass spectrometer (AB SCIEX) after they were separated by UHPLC.

The original data were converted into .mzXML format by ProteoWizard. Then, the XCMS software was used for peak alignment, retention time correction, and extraction of peak area. Accurate mass number matching (<25 ppm) and second stage spectrogram matching were used to identify the metabolite structure, and the laboratory’s self-built database was retrieved. For the data extracted by XCMS, the integrity of the data was first checked. Metabolites with missing values of more than 50% within the group were removed and excluded from subsequent analysis. Extreme values were deleted, and the total peak area of the data was normalized to ensure the parallelism comparison between samples and metabolites.

After pareto-scaling pre-treatment, multivariate statistical analysis was performed on the data. Principal component analysis (PCA) was performed to evaluate population distribution trends among all of the samples. The variable importance in the projection (VIP) value of each variable was calculated by Orthogonal Partial Least Squares Discrimination Analysis (OPLS-DA) to indicate its contribution to the classification. Metabolites with a VIP value > 1 further had a Student’s t-test applied at a univariate level to measure the significance of each metabolite, and *p* values less than 0.05 were considered to be statistically significant. Metabolite fold changes were calculated using a Student’s *t*-test according to the expression levels in the two comparison groups. Using Kyoto Encyclopedia of Genes and Genomes (KEGG, http://www.kegg.jp/ accessed on 8 January 2021) pathway as a unit, Fisher’s exact test was used to analyze and calculate the significance level of metabolite enrichment in each pathway to determine the metabolic and signal transduction pathways that were significantly affected. Z-score was used to standardize peak intensity, and hierarchical clustering analysis of samples based on the Euclidean distance algorithm was implemented in MeV software to reflect the changing trend of metabolites. 

The XCMS software parameters used were as follows. For peak picking, centWave m/z = 25 ppm, peak width = c (10, 60), and prefilter = c (10, 100) were used. For peak grouping, bw = 5, mzwid = 0.025, and minfrac = 0.5 were used.

## 5. Conclusions

In summary, this study demonstrated that UV-B irradiation affects *P. haitanensis* growth and metabolism. Short-term enhanced UV-B irradiation inhibited growth, caused the bleaching of the algal thalli, and increased the contents of total amino acids, soluble sugar, total protein, and MAAs. The results of metabolic analysis provided information on the stress response of *P. haitanensis* to UV-B irradiation. Most of the differential metabolites in *P. haitanensis* were significantly upregulated under UV-B exposure. The differential metabolites of *P. haitanensis* exposed to UV-B radiation were mainly involved in amino acid metabolism, carbohydrate metabolism, glutathione metabolism, and phenylpropane biosynthesis. Our results showed that *P. haitanensis* acts mainly through amino acid metabolism to synthesize UV-absorbing substances (such as phenols and MAAs) to adapt to short-term enhanced UV-B radiation and relies on carbohydrate metabolism and glutathione metabolism to enhance its defense against UV-B radiation.

## Data Availability

The data presented in this study are available in the article and supplementary material.

## Supplementary Materials

Supplementary materials can be found at https://www.mdpi.com/1422-0067/22/4/2180/s1. Figure S1: PCA score diagram of *P. haitanensis* under different UV-B radiation intensities (POS/NEG), Table S1: Relative abundance of all metabolites originally identified in positive ion mode, Table S2: Relative abundance of all metabolites originally identified in negative ion mode, Table S3: Identified differential metabolites of *P. haitanensis* and related information in different comparison groups, Table S4: Relative abundance and statistical analysis of differential metabolites in *P. haitanensis* under different UV-B radiation intensities.
