# Supplementary figures and images for: Effects of Different Short-Term UV-B Radiation Intensities on Metabolic Characteristics of Porphyra haitanensis
Source: Int J Mol Sci. 2021 Feb 22;22(4):2180. doi: 10.3390/ijms22042180 (PMC7927003; doi:10.3390/ijms22042180)

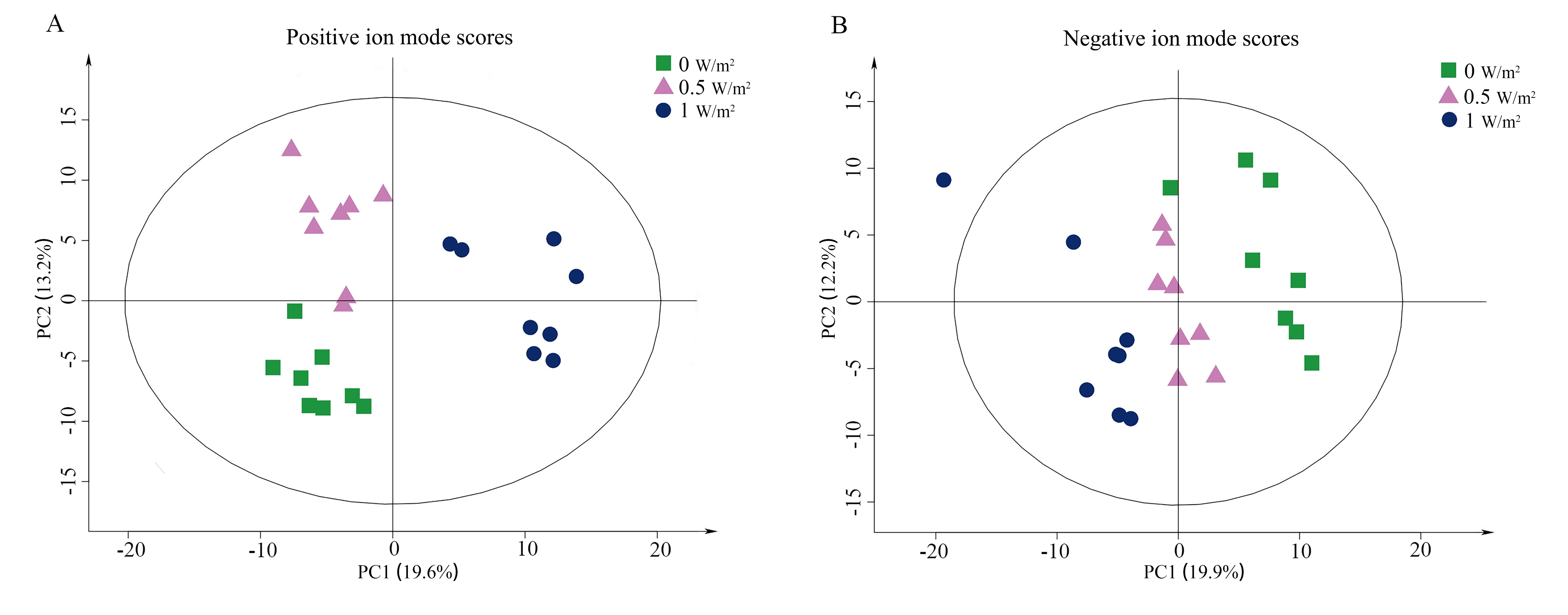

Supplement: Supplementary file 1 [file ijms-22-02180-s001.zip › supplementary files/Supplementary figure S1.tif]
